# Monsanto: partnering for sustainable agriculture

**DOI:** 10.1186/1753-6561-8-S4-O25

**Published:** 2014-10-01

**Authors:** Leigh English

**Affiliations:** 1 Monsanto Company, Chesterfield, MO, USA

## 

Monsanto is driven by a global commitment to sustainable agricultural production. This commitment includes bringing technologies to farmers that improve their lives by helping them be more productive while using fewer resources--leading to a reduced overall effect on the environment. By using this commitment to drive our actions, we believe we are creating a positive impact on our world.

But, we cannot do this alone. That's why, in addition to developing our own products, we also partner and collaborate with others to develop technologies for farmers. We also license our biotech traits and germplasm to other seed companies, giving farmers the opportunity to purchase seed technologies in the brands they choose.

External technology collaborations are a key to our promise of delivering innovative new products in the future. At Monsanto, we are focused on developing collaborative alliances that product lasting benefits for our farmer customers and all involved [1].
